# The effects of telenutrition in overweight and obese adults in a nutritional center in Lima, Peru.

**DOI:** 10.12688/f1000research.53564.1

**Published:** 2021-07-07

**Authors:** Carolina Castrillón Liñan, Jimy Henry Alvarez Mayorga, Michelle Lozada-Urbano

**Affiliations:** 1 Universidad Nacional Mayor de San Marcos, Av Carlos Germán Amezaga # 375, Cercado de Lima, Lima, 15081, Peru; 2 Centro Nutricional Allikay, Centro Nutricional Allikay, Calle de las Artes Norte 269A, San Borja Lima, 15037, Peru; 3 Facultad de Medicina Humana, Universidad Peruana Cayetano Heredia, Av Honorio Delgado # 430, San Martín de Porres, Lima, 15102, Peru; 4 South American Center for Education and Research in Public Health, Universidad Privada Norbert Wiener, Av. Arequipa # 444, Cercado de Lima, 15046, Peru

**Keywords:** Overweight, Obesity, Telemedicine, Waist Circumference, Nutrition Assessment

## Abstract

**Background:** COVID-19 pandemic has been challenging for health services and systems around the world, including Peru.  A viable alternative in the telemedicine field to guarantee patient nutritional care is telenutrition. Telenutrition involves the interactive use of electronic information and telecommunications technologies to implement the nutrition care process with patients at a remote location. Information regarding the experience with this methodology and its potential effect on patients’ nutritional goals, does not exist in Peru. The aim of the study was to report the effect of the evaluation type (telenutrition vs. in-person) on anthropometric parameters weight, body mass index (BMI), waist circumference (WC) and relative fat mass (RFM) in overweight and obese adult patients. **Methods:** This retrospective study included 100 eligible patients in a single nutritional center, from January 2019 to March 2021. **Results:** There was a significant difference in weight, BMI, WC and RFM at the end of the three-month follow-up period, in both evaluation modalities. Patients on the telenutrition group had a mean decrease of 6.80 ± 4.87 cm in their WC, whereas the mean difference observed for the in-person group was 6.74 ± 4.55 cm. There were no statistically significant differences in the changes of any anthropometric parameters when comparing both systems. Reductions were observed in weight (5.93 ± 3.88 kg vs. 4.92 ± 3.29 kg), BMI (2.23 ± 1.39 kg/ m2 vs. 1.83 ± 1.23 kg/ m2), WC (6.80 ± 4.87 cm vs. 6.74 ± 4.55 cm) and RFM (2.43 ± 1.78 vs. 2.63 ± 1.73) in telenutrition and in-person evaluation, respectively. **Conclusions: **Telenutrition may be regarded as an alternative to in-person evaluation offering anthropometric changes and nutritional goals similar to those reported through the in-person modality, in overweight and obese adult people.

## Introduction

Clinical practice has gone through adaptative processes during the COVID-19 pandemic due to isolation and social distancing policies to reduce virus transmission. Telemedicine, a discipline that has been developing for many years, ^1^ provides an alternative to ensure continued patients’ access to health services, while minimizing the risks for health workers. ^2^

Telehealth is the use of electronic information and telecommunication technology to facilitate clinical healthcare and patient education remotely. Whereas telenutrition is defined as a modality of Telehealth that provides an opportunity for a registered dietitian nutritionist to implement patients’ nutritional care remotely. ^3^

Studies in Western China have shown that telemedicine practices are feasible, acceptable, effective and improves health care outcomes, ^4^ by providing the healthcare worker with information about the patient’s surroundings and how homecare is maintained. In New Mexico, USA, telemedicine-related barriers were identified through a survey that included 2016 nutrition professionals. ^5^ In this study, 29% of the professionals reported lack of client interest, 26% reported not having internet access, and 28% mentioned not being able to perform client assessment or monitor activities by this modality. In regard to the benefits of telemedicine, 66% of the professionals stated that it contributed to social distancing compliance, while 50% acknowledged the flexibility in arranging appointments. ^5^

Australian nutrition professionals regard telehealth evaluations as profitable and well-received by patients. They report that this practice improved healthcare access for people with chronic diseases. ^6^

Regarding potential obstacles in telenutrition practices, anthropometric parameters are key for nutritional assessment and are widely discussed when comparing this modality to in-person evaluation. It is known that standard anthropometric assessment involves direct contact, however, there is evidence that self-reported weight and height measurements may have adequate precision. ^7^ Besides, reports state that self-reported WC measurement may be reliable in cases where scales are not available. ^8^^,^^9^ Precise measurement can be achieved by providing video instructions for the patient. ^10^^,^^11^

In Lima, Peru we are facing these challenges as well. In 2020, the Allikay nutritional center started providing virtual appointments for nutritional care to patients, guaranteeing safe conditions based on the COVID-19 pandemic-related recommendations. There is no information regarding the impact of this modality on the patients’ nutritional status measured by anthropometric parameters in Lima, Peru. The aim of this study was to report the effect of the evaluation type (telenutrition vs. in-person) on anthropometric parameters such as weight, body mass index (BMI), waist circumference (WC) and relative fat mass (RFM), in overweight and obese adult patients in a nutritional center in Lima.

## Methods

This is an observational retrospective study that took place between January 2019-March 2021. This study included overweight and obese patients that were assessed by a Registered Dietitian Nutritionist through in-person or virtual appointment, in a nutritional center in Lima.

The samples were obtained through a non-probability convenience sampling. Due to the type of sampling, it is acknowledged that bias is possible and considering this, authors recommend caution when interpreting results in terms of generalization. The final sample size was 100 patients which was arrived by assessing eligibility in every patient evaluated during the established study period (January 2019-March 2021). There were 50 patients evaluated with telenutrition and 50 with in-person assessment. This study included overweight or obese males and females aged 18 years and older, who had both their baseline and three-month assessment data available. Overweight (BMI ≥ 25) and obesity (BMI ≥ 30) was defined as proposed by the World Health Organization (WHO). ^12^ Patients that had bariatric surgery before the recruitment period or were on weight-loss medication at the time of the recruitment, were excluded. All anthropometric parameters were collected twice, at baseline and during the three-month follow-up measurements.

Assessment modalities were either in-person or by telenutrition. The in-person appointment starts with a thorough examination of food habits including two nutritional questionnaires: food consumption frequency and 24-hour dietary recall (Extended data: https://doi.org/10.6084/m9.figshare.14832345.v2). ^13^

Height measurement is self-reported, WC measurement is performed by the nutritionist following standard procedures (middle area between the lower edge of the rib cage and the upper edge of the iliac crest) with a  Lufkin measurement tape. Weight measurement was taken by the Inbody 120 scale. ^14^ Body fat percentage was calculated with the use of the following RFM formula described by Woolcott OO and Bergman RN: ^15^

Male adults: 64−(20 × (height cm/waist circumference (cm))

RFM ≥ 22.8 for obesity in males.

Female adults: 76−(20 × (height cm/waist circumference cm))

RFM ≥ 33.9 for obesity in females.

Once the anthropometric data is taken, the nutritionist creates a personalized nutritional plan adapted to the patients’ nutritional status, food habits and preferences. The follow-up and monitoring are performed through e-mail or by the multimedia messaging application (WhatsApp), once a week. Standard follow-up appointments are performed every 20 days. The three-month follow up assessment includes WC, weight measurements and RFM.

**Figure 1.**
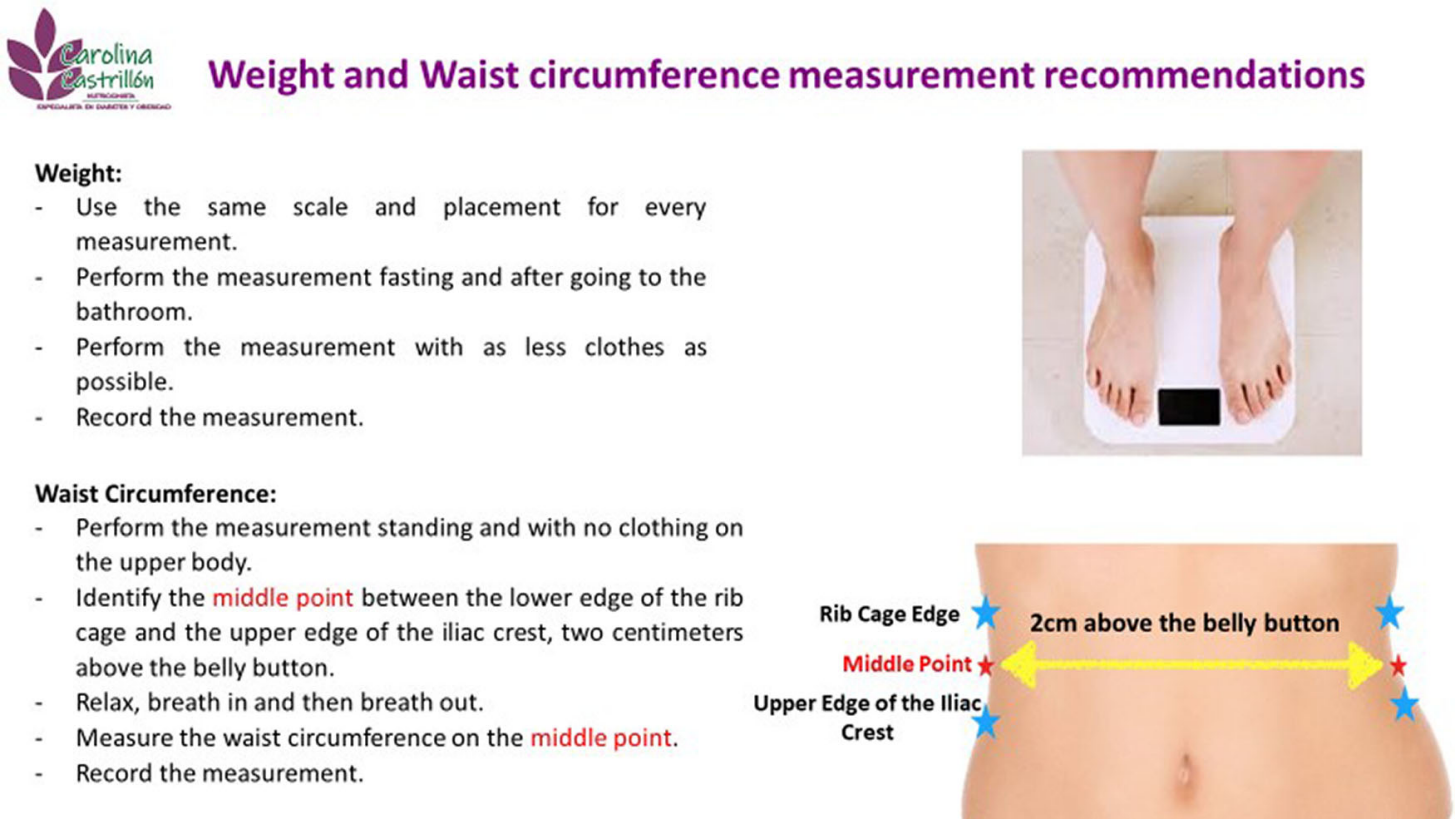
Infographic for subjects.

Source: created por Castrillón-Liñán C. based on Aguilar Esenarro L, Contreras Rojas M, Del Canto y Dorador J, Vílchez Dávila W. Guía técnica para la valoración nutricional antropométrica de la persona adulta [Internet]. Instituto Nacional de Salud; 2012. Disponible en: https://repositorio.ins.gob.pe/handle/INS/225.

Telenutrition assessment starts one day before the actual appointment by sending an infographic that includes fasting anthropometric measurement instructions to the patient based on the local recommendations. ^14^ This is done in order to standardize weight and WC measurements for remote assessments ( Figure 1). During the appointment, height value is self-reported and the nutritionist requests the self-measured values (weight and WC) for the RFM calculation. Following this, the nutritional plan adapted to the patients’ nutritional status, food habits and preferences is sent to the patient by e-mail. The follow-up and monitoring are once a week and it is done by e-mail or WhatsApp. Standard follow-up appointments are performed every 20 days. The three-month follow -up assessment includes self-reported WC, weight measurements and RFM calculation.

### Data analysis

Data analysis was performed with the use of Stata/SE 12.0 for Windows ( https://www.stata.com/). Continuous variables were examined for outliers with graphic (box plot) and analytic techniques (interquartile ranges). Normality was assessed with graphic (histograms) and analytic techniques for skewness and kurtosis (Shapiro Wilk test). For univariate analysis, continuous variables were described with means and standard deviations or medians and ranges. Categorical variables were reported as frequencies. For bivariate analysis, baseline and follow-up continuous variables were compared with paired-samples t-test or Wilcoxon Rank-sum test. Telenutrition and in-person continuous variable values were compared with independent samples t-test or U Mann-Whitney test. Categorical variables were compared with the chi-square test. P-value < 0.05 is considered statistically significant.

Dataset created and analyzed for the study is available as underlying data: https://doi.org/10.6084/m9.figshare.14710296.v1. ^16^

### Ethical considerations

This study was conducted according to the Declaration of Helsinki principles. Patients were deidentified by being assigned to code numbers, without any reference to the patient information, therefore keeping the data confidential. Patient information was only accessible to the Registered Dietitian Nutritionist who evaluated the patients. Informed consent was not needed for this retrospective study as the data had been anonymized and this was confirmed by the Ethics Committee which approved the study. Ethical approval was obtained from the Institutional Ethics Committee for Research of the Norbert Wiener University with Exp. No 526-2021.

## Results

Mean baseline BMI was in the obesity range (31.91 ± 5.53 and 30.36 ± 4.35 kg/m ^2^ for telenutrition and in-person group respectively), while the mean final BMI was in the overweight range (29.68 ± 5.02 and 28.53 ± 4.12 kg/m ^2^ for telenutrition and in-person group respectively) ( Table 1).

**Table 1.** Clinical and anthropometric characteristics according to the assessment group.

Variables	Telenutrition (n = 50)	In-person assessment (n = 50)	p
Sex Men Women	8 (16%) 42 (84%)	15 (30%) 35 (70%)	0.096
Age (years)	40.66 ± 12.69	38.18 ± 10.17	0.2835
Height (cm)	162.72 ± 7.51	164.32 ± 8.59	0.3585
Baseline weight (kg)	84.93 ±18.21	82.22 ± 14.60	0.6994
Baseline BMI (kg/m ^2^)	31.91 ± 5.53	30.36 ± 4.35	0.1160
Baseline WC (cm)	100.14 ± 14.40	96.30 ± 11.82	0.1482
Baseline RFM	41.03 ± 5.30	37.85 ± 6.53	0.0088
Follow-up weight (kg)	79.00 ± 16.62	77.30 ± 13.95	0.9258
Follow-up BMI (kg/m ^2^)	29.68 ± 5.02	28.53 ± 4.12	0.2095
Follow-up WC (cm)	93.34 ± 13.39	89.56 ± 11.30	0.1301
Follow-up RFM	38.60 ± 5.53	35.23 ± 6.73	0.0073

BMI: body mass index, WC: waist circumference, RFM: relative fat mass. P < 0.05.

The baseline and three-month follow-up weight, BMI and WC values were not statistically different between the two assessment groups. However, significant differences were observed in the mean baseline RFM (41.03 ± 5.30 vs. 37.85 ± 6.53) and mean three-month follow-up RFM (38.60 ± 5.53 vs 35.23 ± 6.73), between the assessment groups ( Table 1).

Significant differences between baseline and three-month follow -up weight, BMI, WC and RFM within the assessment groups (p = 0.0000) were identified, the greatest difference reported was in WC, which decreased by 6.80 ± 4.87 cm and 6.74 ± 4.55 cm in telenutrition and in-person assessment, respectively ( Tables 2 and 3).

**Table 2.** Anthropometric parameters for subjects evaluated by Telenutrition (n = 50).

Variables	Baseline assessment	Follow-up assessment	Difference	p
Weight (kg)	84.93 ±18.21	79.00 ± 16.62	5.93 ± 3.88	0.0000
BMI (kg/m ^2^)	31.91 ± 5.53	29.68 ± 5.02	2.23 ± 1.39	0.0000
WC (cm)	100.14 ± 14.40	93.34 ± 13.39	6.80 ± 4.87	0.0000
RFM	41.03 ± 5.30	38.60 ± 5.53	2.43 ± 1.78	0.0000

BMI: body mass index, WC: waist circumference, RFM: relative fat mass. P < 0.05.

**Table 3.** Anthropometric parameters for subjects evaluated by in-person assessment (n = 50).

Variables	Baseline assessment	Follow-up assessment	Difference	p
Weight (kg)	82.22 ± 14.60	77.30 ± 13.95	4.92 ±3.29	0.0000
BMI (kg/m ^2^)	30.36 ± 4.35	28.53 ± 4.12	1.83 ± 1.23	0.0000
WC (cm)	96.30 ± 11.82	89.56 ± 11.30	6.74 ± 4.55	0.0000
RFM	37.85 ± 6.53	35.23 ± 6.73	2.63 ± 1.73	0.0000

BMI: body mass index, WC: waist circumference, RFM: relative fat mass. P < 0.05.

In regard to the change in the magnitude of the anthropometric parameter values between the assessment groups, no significant differences were identified for any parameter. Weight (5.93 kg and 4.92 kg), BMI (2.23 kg/m ^2^ and 1.83 kg/m ^2^), WC (6.80 cm and 6.74 cm), and RFM (2.43 and 2.63) reductions were similar in both telenutrition and in-person assessment, respectively ( Table 4).

**Table 4.** Difference in anthropometric parameters according to assessment group.

Variables	Telenutrition change	In-person assessment change	p
Weight (kg)	5.93 ± 3.88	4.92 ±3.29	0.1641
BMI (kg/m ^2^)	2.23 ± 1.39	1.83 ± 1.23	0.1265
WC (cm)	6.80 ± 4.87	6.74 ± 4.55	0.9510
RFM	2.43 ± 1.78	2.63 ± 1.73	0.5741

BMI: body mass index, WC: waist circumference, RFM: relative fat mass. P < 0.05.

## Discussion

This study was designed to examine variations in the anthropometric parameters of overweight and obese adults treated in a private nutritional center where telenutrition was additionally implemented. The results showed that weight loss was statistically significant in both groups, with no difference in the variation by the three-month follow-up evaluation. Weight loss is a key factor in reducing non-communicable disease risk and COVID-19 complications, the fact that this nutritional goal is achieved through both assessment modalities, offers the patient a viable alternative for health maintenance.

Significant changes in weight, WC, BMI and RFM were observed within each assessment modality in our study. However, comparing the changes in these anthropometric measures between these two assessment modalities, no significant difference in these values was identified. Kuzmar IE et al., reported similar results, with no significant differences in weight loss, BMI and waist-to-height ratio (WHtR), when comparing the in-person assessment to telenutrition, in obese women. ^17^

Other studies have identified significant changes in anthropometric parameters, such as Huang et al., who had assessed overweight and obese patients with non-communicable diseases by using telenutrition and observed a significant BMI decrease. ^18^ Whereas Beleigoli et al., used a web-based software with patient feedback for 24 weeks in overweight and obese patients in comparison to non-technological interventions to assess weight loss and lifestyle changes, and indicated improvement in food consumption habits, user adherence and significant weight loss. ^19^ Likewise, Ventura Marra et al., in a randomized study in cardiovascular patients observed a significant weight loss in a similar follow-up period of 12 weeks, 4.92 ± 3.29 kg and 5.93 ± 3.88 kg for in-person modality and telenutrition, respectively. ^20^ In five American clinics, a randomized study examined pregnant women to prevent excessive weight gain and promote healthy behavior by comparing telehealth strategies to traditional assessment. Pregnant women assessed traditionally showed an average weekly gestational weight loss of 0.26kg, in comparison to 0.32kg in the telehealth group (the mean difference between the two groups was 0.07 kg per week, CI 95%: −0.09 to −0.04). ^21^

A study that compared video conference health coaching with a focus on physical activity and weight management, to in-person modality for adults with high BMI, showed that the intervention group achieved a significantly greater weight loss (8.23 ± 4.5 kg), ^22^ within 12 weeks.

Another telehealth program for weight loss that used video conferencing for 12 weeks showed a significant difference between the intervention and control groups in body weight (7.16 ± 4.4 vs. 1.5 ± 4.1%, respectively). The significant weight loss was achieved in nine out of 13 individuals (69.2%) in the intervention group compared to one in 12 (8%) in the control group. ^23^

It is important to emphasize that in our study, the greatest decrease obtained in the telenutrition assessment was for WC (mean difference 6.80 ± 4.87 cm), which is an anthropometric index significantly associated with increased risk to cardiovascular and metabolic diseases, as reported in the literature. ^24^^-^^27^

Additionally, this study has included RFM which as an anthropometric parameter has not been extensively examined in Lima, Peru. RFM, is a valuable tool for corporal composition evaluation, ^15^^,^^28^ and for being a great predictor of dyslipidemia and metabolic syndrome. ^29^ In our study, RFM showed also a significant decrease in both modalities (2.43 ± 1.78 telenutrition vs 2.63 ± 1.73 in-person).

This study has some limitations, consider the observational and retrospective nature of the study. There may be other variables, such as physical activity, lifestyle changes, previous nutritional consultation or weight-loss concomitant medications, which have not been assessed that could act as confounders. The study was conducted in a private nutritional center and through a convenience sampling strategy which may impact representativity. The follow-up period was three months, so either stabilization or further variations of the assessed parameters may take place which could be observed with longer follow-up periods. Future prospective studies could assess the long-term impact of telenutrition in anthropometric parameters and nutritional health in patients.

## Conclusion

In the time of pandemic, telenutrition has become a valuable alternative to nutritional care by reducing the transmission risk through social distancing practices. Telenutrition may well be regarded as a useful tool for current situations such as the COVID-19 pandemic, by offering similar outcomes to those reported as the in-person assessments, while providing ongoing nutritional support to overweight and obese adults in times of isolation and social distancing. Health care providers should attempt to adapt their processes to fulfil the patients’ health demands, in order to prevent excessive weight gain and its related comorbidities through interventions like telenutrition.

## Data availability statement

### Underlying data

Figshare: The effects of telenutrition in overweight and obese adults in a nutritional center in Lima, Peru.

DOI: https://doi.org/10.6084/m9.figshare.14710296.v1. ^16^

The project contains the following underlying data:

Database: The data includes baseline and 3-month follow up demographic and anthropometric parameters for overweight and obese adults according to assessment modality (telenutrition vs in-person).

### Extended data

*Figshare:* The effects of telenutrition in overweight and obese adults in a nutritional center in Lima, Peru.

DOI: https://doi.org/10.6084/m9.figshare.14832345.v2. ^13^

This project contains the following extended data:

File 1: Consumption Frequency Questionnaire

File 2: 24-hour Dietary Recall Questionnaire

Data are available under the terms of the Creative Commons Zero “No rights reserved” data waiver (CC0 1.0 Public domain dedication): https://creativecommons.org/publicdomain/zero/1.0/.
