# Supplementary material for: The effects of telenutrition in overweight and obese adults in a nutritional center in Lima, Peru
Source: F1000Res. 2021 Nov 15;10:545. Originally published 2021 Jul 7. [Version 2] doi: 10.12688/f1000research.53564.2 (PMC8666983; doi:10.12688/f1000research.53564.2)
Supplement: Peer review history

# Peer review: Reviewer response for version 2

**DOI:** 10.5256/f1000research.78819.r100227

**Published:** 2021-12-07

**Authors:** Katherine Curi Quinto

**Affiliations:** 1 Nutrition Research Institute, Cuernavaca, Mexico

All my previous comments was considered by the authors. I have no further comments, and I believe this article is a key contribution to the field of nutrition counseling.

Is the work clearly and accurately presented and does it cite the current literature?

Yes

If applicable, is the statistical analysis and its interpretation appropriate?

Partly

Are all the source data underlying the results available to ensure full reproducibility?

Yes

Is the study design appropriate and is the work technically sound?

Partly

Are the conclusions drawn adequately supported by the results?

Partly

Are sufficient details of methods and analysis provided to allow replication by others?

Partly

Reviewer Expertise:

Nutrition epidemiology

I confirm that I have read this submission and believe that I have an appropriate level of expertise to confirm that it is of an acceptable scientific standard.

# Peer review: Reviewer response for version 1

**DOI:** 10.5256/f1000research.56963.r92606

**Published:** 2021-09-23

**Authors:** Katherine Curi Quinto

**Affiliations:** 1 Nutrition Research Institute, Cuernavaca, Mexico

**Overall comment:**

 This manuscript is an important contribution to inform current practice/strategies and intervention to promote a better nutritional and health status in the current context of the COVID-19 pandemic, although it could be sustained over time as a new strategy in the nutritional practice.  

 Given this importance, I highlight the following aspects to clarify in the manuscript. This will help to improve the scientific quality of the document:

  

1. **Abstract: **Add the statistical analysis in methods and specify the group of comparison for the last results presented in brackets (weight, BMI, WC, RFM.)
2. **In the overall manuscript: **Explain why the design of the study is retrospective? according to the flow of this study, it seems a quasi-experimental study with "prospective" repeated measurements over the time of the intervention (longitudinal), unless you want to present as a secondary data analysis which could be also adequate mainly because you did not have the inform consent of the participants.  If the latter is your case, you must make this clarification in your manuscript.
3. **In the methods: **
  a) Explain how you did the allocation of the 50 participants per each group of intervention and add the inclusion criteria for eligibility. 
  b) Give more detailed information about the intervention itself: characteristics of the nutritional plan, how and what indicators did you follow & monitor? in case you did for both groups. You could also specify what kind of messages/mail/text, etc. you included.
  c) In data analysis, add which values ​​you considered outliers and report: how many and how you handled the outliers (based on your results, it appears there were no outliers).
  d) Since this is a convenience sample, it will be contributed to the internal validity of your study if you add information on key sociodemographic factors such as level of education. In case you did not collect this information, this must be reported as a limitation and explain why the impact in the results you could be are reporting.
4. **In the results:**
  a) In the footnotes of the tables indicate the test from which you obtained the p-value.
  b) The tables repeat information. It would be better if you avoid these types of repetitions. As a suggestion, you could present in Table 1 only the initial characteristics. Tables 2 and 3 can be joined without presenting the differences between the groups. The reader has enough information to know the differences. Then you could keep table 4 as is.
5. ** **
**In the discussion:**
  a) The discussion needs to be stronger. Add a possible explanation of your results and discuss it in the context of the previous evidence related to the comparison of traditional and telenutrition intervention.  The explanation could be in terms of differences or advantages/disadvantages of the method used and why it could influence the results, or it could be for the difference in the intervention itself (frequency, intensity, different ways to monitor, the same nutritionist give the same intervention, some baseline sociodemographic characteristics such as literacy, etc.).
  b) The greater differences in WC in the telenutrition group could be associated with a measurement error, so how do you control that? Can you make cross-validation with the self-reported weight and BMI data? It is more likely that people with weight loss also have a reduction in WC and other parameters. Discuss a little more about this finding that you highlighted in the manuscript.
  c)  Add the clinical implication of the RFM. This is a non-common measure, as you presented in the manuscript, but there is little information about its relevance in the clinical nutrition field. Moreover, consider adding information about the validation in the Peruvian population of the equation you used to estimate the RFM.
  d) In the limitation specify how the no inclusion of confounders could affect your results.
  e) Consider highlighting the strengths of your study, I think it has several. You provide important material in terms of methods to the assessment of the Food intake as well as the Food habits.

Is the work clearly and accurately presented and does it cite the current literature?

Yes

If applicable, is the statistical analysis and its interpretation appropriate?

Partly

Are all the source data underlying the results available to ensure full reproducibility?

Yes

Is the study design appropriate and is the work technically sound?

Partly

Are the conclusions drawn adequately supported by the results?

Partly

Are sufficient details of methods and analysis provided to allow replication by others?

Partly

Reviewer Expertise:

Nutrition epidemiology

I confirm that I have read this submission and believe that I have an appropriate level of expertise to confirm that it is of an acceptable scientific standard, however I have significant reservations, as outlined above.

# Response

**Published:** 2021-11-11

**Authors:** Michelle Lozada-Urbano

**Affiliations:** Universidad Privada Norbert Wiener, Peru

Suggestion acknowledged and clarifications described in the Amendments from Version 1 and specified on the manuscript in detail.

# Peer review: Reviewer response for version 1

**DOI:** 10.5256/f1000research.56963.r93552

**Published:** 2021-09-20

**Authors:** María Del Mar Morales Hernández

**Affiliations:** 1 Internal Medicine, University of Florida College of Medicine, Gainesville, FL, USA

Clinically relevant retrospective study about the effects of telenutrition in obese and overweight patients in Lima, Peru. The study includes both study and control groups. The authors used easy-to-follow questionnaires and imaging to guide patient's nutritional care. Also provided good weekly follow-up for patients with aid of the technology. Would have liked to see an example of the nutritional plan utilized and if those were similar to in person vs telemedicine patients. Having no statistical significance in changes on anthropometric parameters between groups will suggest that patients could still receive benefits of nutritional care during the pandemic and expect to have similar results as in person encounters in their weight control. This study showed changes in weight, waist circumference, BMI, and RFM.  Improvement of nutrition is key to decrease disease burden in the times of COVID 19 pandemic and this study adds to the usefulness of telemedicine and in this specific case telenutrition to help address risk factors during these challenging times.

Is the work clearly and accurately presented and does it cite the current literature?

Yes

If applicable, is the statistical analysis and its interpretation appropriate?

Yes

Are all the source data underlying the results available to ensure full reproducibility?

Yes

Is the study design appropriate and is the work technically sound?

Yes

Are the conclusions drawn adequately supported by the results?

Yes

Are sufficient details of methods and analysis provided to allow replication by others?

Yes

Reviewer Expertise:

Endocrinology disorders, diabetes technology

I confirm that I have read this submission and believe that I have an appropriate level of expertise to confirm that it is of an acceptable scientific standard.
